# A Case of Psychosis and Renal Failure Associated with Excessive Energy Drink Consumption

**DOI:** 10.1155/2019/3954161

**Published:** 2019-07-24

**Authors:** D. Kelsey, A. J. Berry, R. A. Swain, S. Lorenz

**Affiliations:** ^1^North London Forensic Service, Barnet, Enfield and Haringey Mental Health Trust, Chase Farm Hospital, London EN2 8JL, UK; ^2^National Hospital for Neurology and Neurosurgery, Queen Square, London, WC1N 3BG, UK; ^3^Camden and Islington NHS Foundation Trust, St. Pancras Hospital, 4 St. Pancras Way, London NW1 0PE, UK; ^4^Consultant Psychiatrist, Barnet, Enfield and Haringey Mental Health Trust, St. Ann's Hospital, London N15 3TH, UK

## Abstract

Energy drinks are nonalcoholic beverages that are widely consumed in the general population, and worldwide usage is increasing. The main stimulant component of energy drinks is typically caffeine. Few case reports exist that link energy drink consumption to psychosis, and similarly few reports exist that associate energy drink consumption with acute renal failure. We present a patient who simultaneously developed psychosis and acute renal failure associated with excessive energy drink consumption. The patient required haemodialysis, and his psychosis resolved on cessation of energy drinks and a brief course of antipsychotic medication. We perform a review of similar cases where excessive caffeinated energy drink consumption has been linked to psychosis or acute renal failure. To our knowledge, this is the first case report describing both renal failure and psychosis occurring simultaneously in a patient. Recognising the spectrum of disorders associated with excessive energy drink consumption is vital for both physicians and psychiatrists, as this has important implications for both prognosis and treatment.

## 1. Introduction

Energy drinks (ED) are nonalcoholic beverages that contain, amongst other ingredients, the stimulant, caffeine, and the neuroinhibitory amino acid, taurine. They are widely consumed in the general population for their purported enhancing effects on cognition and physical performance, and their use is increasing. In 2011, a study commissioned by the European Food Safety Authority (EFSA) estimated the consumption of energy drinks within the EU at 30% of adults and 68% of adolescents. Global sales in 2012 were estimated to total roughly $12 billion [1]. Caffeine (1,3,7-trimethylxanthine) has been consumed for millennia and is the most used psychotropic substance in the world. Caffeine is found in a wide variety of foods and beverages, and the EFSA state that a “standard” 250 ml energy drink (referenced as the most popular brand) contains a caffeine concentration of 0.32mg/ml [2]. In reality, ED vary in caffeine content, dependent on concentration and volume [3]. Furthermore, ED typically contain numerous additives such as D-glucurono-*γ*-lactone, taurine, guarana, and ginseng. A recent narrative review of ED consumption commissioned by the World Health Organisation highlighted numerous caffeine-associated risks and adverse health outcomes, as well as the need for further research into the long-term effects of ED consumption on global health [4]. Caffeine is thought to exert its stimulant effects through antagonism of adenosine 2A receptors which in turn may result in increased central nervous system dopaminergic activity [5]. The association between caffeine and mental disorder is well documented. Psychiatric symptomatology caused by excessive caffeine consumption may range from anxiety and dysphoria [6, 7] to mania and psychosis [8–11].

Moderate caffeine intake may have beneficial effects on attention, memory encoding, and mood [12], though its abuse has been reported in differing patient groups. One recent systematic review identified caffeine as the second most abused substance after tobacco in those with eating disorders [13], and cases of athletes abusing caffeine for its performance-sustaining effects have also been reported [14]. Amongst body builders in particular, caffeine abuse may be additionally associated with the presence of body dysmorphic disorder [14]. It has also been proposed that consumption of high doses of caffeine may be used to self-medicate depression [15].

## 2. The Case

A British male in his 30s of south Asian ethnicity was arrested for offences of criminal damage and possession of a bladed article. At arrest, the patient required mechanical restraint and was transferred to a local emergency department for further assessment, where 2 mg of lorazepam was administered intramuscularly. Examination revealed no injuries, and he was discharged back to police custody. He was then involuntarily detained to a psychiatric ward for further investigation and treatment. During initial psychiatric assessment on the ward, the patient exhibited agitation and anxiety and revealed he had been carrying the knife due to delusions of persecution. No passivity phenomena, thought alienation, or perceptual abnormalities were elicited during the interview.

The patient, a shift worker, disclosed drinking up to 12 250 ml cans of Red Bull (a widely available ED) per day, consuming an estimated daily total of 960 mg caffeine in the days preceding his admission, to keep up with the demands of his job. On the day of his arrest he had drunk 7 units of alcohol and denied illicit drug use. There was no history suggestive of alcohol- or illicit drug-dependence.

Social history revealed that the patient had separated from his wife and returned to living with his mother in the preceding month. Family members had not noticed any significant deterioration in mental state during this time. His behaviour leading to the current presentation was considered out of character.

The patient's past psychiatric history consisted of one episode of low mood 12 years prior, and one deliberate overdose a year later. On both occasions, no referral to psychiatric services was made, and no treatments were initiated.

There was no family history of mental or physical illness.

The patient complained of back pain soon after admission to the psychiatric unit. Physical examination findings and vital signs were within normal limits. Lumbar and sacral x-rays showed no bony pathology. Two days following admission the patient had worsening abdominal pain and started vomiting. Blood samples showed a creatinine (Cr) of 1205 *μ*mol/L (normal range 59-104*μ*mol/L), estimated glomerular filtration rate (eGFR) of 4 mL/minute (normal range > 90 ml/min) consistent with an acute kidney injury (AKI) of grade 3 severity according to Kidney Diseases Improving Global Outcomes (KDIGO) classification, creatine kinase (CK) of 3336 U/L (normal range 40-320 U/L), and C-reactive protein (CRP) of 38.8 mg/L** (**normal range <10mg/L). The remainder of the blood results (including electrolytes, serum calcium, and liver function tests) were within normal limits. The patient had received two doses of 5 mg olanzapine, four doses of 400 mg ibuprofen, four doses of 10 mg dihydrocodeine/500mg paracetamol, and 50 mg of cyclizine during his admission up to that point.

The patient was then transferred to an acute hospital. The patient reported that he had passed little urine following his arrest. Examination revealed right flank tenderness. There was no fever, rash, or haemoptysis. Venous blood gas revealed an acidosis, with pH of 7.27 (normal range 7.31-7.41), HCO3 of 18 mEq/L, and lactate of 1.9mmol/L [normal range <2mmol/L]. Urinalysis showed microhaematuria, proteinuria, and leucocytes. Chest x-ray was unremarkable, and noncontrast computed tomography (CT) of the abdomen and pelvis revealed normal appearances of the kidneys and no intra-abdominal abnormalities. Blood tests were negative for rheumatoid factor, antinuclear antibodies (ANA), antiextractable nuclear antigen (Anti-ENA), antiglomerular basement membrane (Anti-GBM), and myeloperoxidase antibodies (MPO-ANCA). Proteinase 3 antibodies (PR3-ANCA) were borderline positive at 6 units/mL, but not felt to be of clinical significance. Serology was negative for human immunodeficiency virus, syphilis, and hepatitis A, B, and C. Serum immunoglobulins and complement proteins C3 & C4 were within normal limits

**Figure 1:**
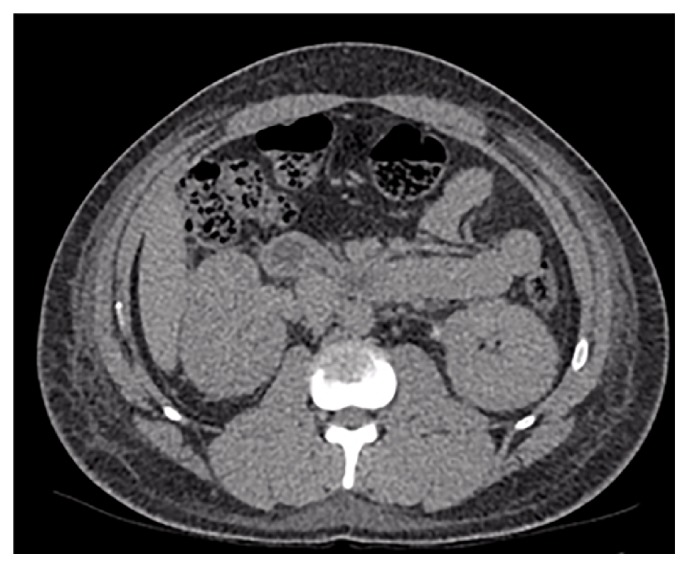
Noncontrast CT section of the abdomen, showing subcutaneous oedema.

A working diagnosis of tubulointerstitial nephritis secondary to toxin ingestion was considered. The elevated CK was thought due to a combination of restraint, psychomotor agitation, and coexistent reduced renal clearance. He remained acutely agitated and was unable to tolerate a renal biopsy. Repeat noncontrast CT of the abdomen and pelvis demonstrated widespread subcutaneous oedema throughout the abdomen and pelvis likely secondary to acute renal impairment (Figure 1). MRI head revealed no intracranial abnormalities.

The patient underwent haemodialysis and by the 11th day of his admission his creatinine resolved to 159*μ*mol/. Hospital psychiatric review revealed some irritability and impulsivity persisted. He was returned to the psychiatric unit 17 days after his initial presentation. Mental state examination revealed no evidence of psychosis, though the patient appeared mildly elated. Olanzapine was increased to 10 mg nocte and modified-release sodium valproate was introduced.

On discharge from the psychiatric unit, mental state examination revealed no signs of psychosis, but a mildly expansive mood persisted. He was taking 20 mg olanzapine and 600 mg sodium valproate daily at point of discharge.

Two weeks after discharge the patient was reviewed in the renal outpatient clinic where creatinine was 111*μ*mol/L, and urinary albumin:creatinine ratio was within normal limits. Renal function has since remained stable and there were no subsequent admissions to hospital 6 months after discharge.

On psychiatric follow-up, two months after discharge, he displayed no psychotic or affective symptoms. He had discontinued all psychotropic medication a week following discharge (against medical advice). He no longer consumed energy drinks and reported to have not taken illicit substances in that time. Telephone follow-up six months following discharge revealed no concerns regarding his mental state. The patient has since been discharged from psychiatric follow-up, and in light of this we have been unable to obtain specific written consent from the patient for the case report. As such, we have omitted specific details about the patient's age and occupation in order to suitably preserve anonymity.

## 3. Discussion

This patient was diagnosed with caffeine-related psychosis associated with excessive consumption of energy drinks, in addition to energy drink-related acute kidney injury (AKI). Whilst there are a small number of case reports of caffeine-related psychosis and energy drink-related AKI, this is the first report describing both occurring simultaneously.

There are currently no operationalised criteria for a diagnosis of caffeine-related psychosis, though some have proposed psychosis may occur when caffeine concentrations reach 10-15 mg/kg [16]. ICD- 10 provides no specific guidance on diagnosis of caffeine-related psychiatric disturbance, but the reported case would meet criteria for the diagnosis “mental and behavioural disorders due to use of other stimulants, including caffeine (F15.00).” The Diagnostic and Statistical Manual for Mental Disorders 5th Edition (DSM-V) proposes 4 caffeine-related syndromes, with this case fulfilling criteria for a diagnosis of “other caffeine-induced disorders.” The DSM-V expounds that “these caffeine-induced disorders are diagnosed instead of caffeine intoxication or caffeine withdrawal only when the symptoms are sufficiently severe to warrant independent clinical attention” and notes that similar disorders such as “caffeine-induced anxiety” disorder would also come under this diagnostic category.

Caffeine intoxication (sometimes referred to as “caffeinism”) shares many physiological and psychological features with anxiety disorders and typically occurs when daily intake of caffeine is over 1000 mg [6, 7]. Death has been described from caffeine toxicity, with potentially lethal doses being associated with plasma concentrations of 70 mg/L and above. A recent review of deaths associated with caffeine use highlighted a number of suicides where caffeine toxicity has been implicated (usually with consumption of caffeine-containing pills, rather than energy drinks) [14].

Caffeine is thought to exert its stimulant effects via antagonism of adenosine A2A receptors (A2AR), and stimulant effects have been reported with other A2AR antagonists. Whilst the exact mechanism remains yet to be fully elucidated, pharmacological studies suggest this may be due to caffeine's antagonism of A2AR leading to inhibition of internalisation of membrane-bound A2AR- and Dopamine D2 receptor heteromers within striatal neurons [5].

A small number of case reports describe the emergence of psychosis following excessive caffeinated drink consumption in those without previously diagnosed psychotic disorders, with the first clear case being reported in 1936 [8]. More recently, Hernandez-Huerta et al.[9], Cruzado et al. [10], and Sharma et al. [11] have described 3 cases of psychosis arising de novo following excessive caffeine intake. The ages of the 3 patients range from 18 to 32, and daily caffeine intake ranges from 480 mg to 1300 mg per day. All 3 cases presented with agitation, often requiring use of sedative and antipsychotic medication for brief periods. In all cases, stopping caffeine consumption was associated with resolution of psychotic symptoms. In Cruzado et al.'s case, recurrence of psychotic symptoms was temporally associated with the patient subsequently increasing intake of caffeine after initial recovery and resolved again following reduction in caffeine intake. In all cases, psychotic symptoms were short lived (with symptoms typically lasting under 1 month) and appeared to be associated with a good prognosis at follow-up (follow-up periods ranged between 6 weeks and 2 years). Intercurrent life stressors (particularly work-related stress) were frequently associated with the high consumption of caffeine/energy drinks in the reported cases of caffeine-associated psychosis [9, 10].

The contributions that psychological stressors and excessive caffeine use may play in the development of psychotic states are important to consider, and clinicians should bear in mind that escalating caffeine use may be an indicator of an individual experiencing considerable stress. A study in a nonclinical population demonstrated those who self-rated as “most stressed,” and also had a high intake of caffeine (>200mg/day), were significantly more likely to falsely identify sounds during an experimental paradigm, suggestive of a degree of “hallucination proneness” in this group [17].

Pharmacological evidence of caffeine's effects on striatal dopaminergic activity suggests a biologically plausible mechanism for the generation of aberrant perceptual experiences and psychotic symptoms [5]. Furthermore, a number of studies indicate caffeine use increases psychotic symptoms in patients with established diagnoses of psychotic disorders [6, 18–20].

We cannot exclude the possibility that our case, and the similar cases reported, may have an underlying vulnerability to the development of an enduring psychotic syndrome, and it is notable that in, Cruzado's case report, there was a strong family history of affective illness [10].

We are aware of three reported cases of AKI associated with excessive energy drink consumption, with ages ranging from 17 to 40, and with daily caffeine intake amongst cases estimated to range from 780 mg to 1140 mg [21, 22]. Additionally it was reported that one patient was primarily ingesting Guarana (Paullinia cupana, a plant producing a caffeine-containing seed, which is commonly added to energy drinks), though the amount drunk was not quantified [23]. Two of the three patients required renal replacement therapy, whilst Greene et al. report that renal function normalised 2 weeks following cessation of energy drink consumption alone. None of these cases appear to have been associated with psychosis, but Greene et al. describe their patient as showing signs of hyperkinesis and agitation. Renal biopsy results were available for one patient, showing acute tubular necrosis, though this patient was also taking nonsteroidal anti-inflammatory drugs concomitantly, which is also associated with acute tubular necrosis [23]. Schoffl et al. reported biochemical evidence of acute tubular necrosis, and the patient was reported to also have a raised CK at the time of renal failure [21].

It is important for the psychiatrist to appreciate the potential association between excessive energy drink consumption and acute renal failure, as this has important implications for the treatment of psychiatric syndromes. Choice of antipsychotic treatment should be carefully considered in those with renal impairment, as there is considerable variability between the extents to which renal impairment may affect pharmacokinetics of individual antipsychotic medications. For example, aripiprazole and ziprasidone are minimally excreted by the kidneys, whereas amisulpride and paliperidone primarily undergo renal excretion and may be associated with an increased risk of toxicity in those with renal impairment [24]. Clozapine and olanzapine should also be used with caution, due to their propensity to elevate CK levels (though the likelihood of this causing AKI in isolation is low, but theoretically may compound preexisting renal impairment) [25].

Excessive energy drink consumption has been reported to complicate the treatment of bipolar affective disorder, and it is notable that renal impairment associated with excessive energy drink intake will increase risk of the development of lithium toxicity [18]. Caffeine is metabolised by the CYP1A2 cytochrome P450 enzyme, acting additionally as a CYP1A2 autoinhibitor, which has theoretical implications for the choice of antipsychotic treatment in caffeine-related psychosis (as clozapine, haloperidol, and olanzapine are metabolised by this enzyme) [24]. The metabolism of caffeine produces 14 metabolites, some of which also possess A2AR antagonist properties, and some of which also undergo subsequent renal excretion [26].

This case highlights two separate consequences of excessive energy drink consumption that psychiatrists and physicians should be aware of, as they have implications for safe treatment and prognosis. Enquiry into caffeine intake and use of energy drinks may often be overlooked in psychiatric assessments. Due to its widespread use in the population, the potential for abuse, and the diverse potential psychiatric sequelae, caffeine consumption should be specifically assessed in the clinical history.
